# Investigating the Effect of Fly Ash Addition on the Metallurgical and Mechanical Behavior of Al-Si-Mg-Cu Alloy for Engine Cylinder Head Application

**DOI:** 10.3390/ma15155462

**Published:** 2022-08-08

**Authors:** Karthik Venkitraman Shankar, Jan Jezierski, Vaira Vignesh Ramalingam, Devaprasad Padmakumar, Midun Raj Leena, Amal, Gokul Reghunath, Rakesh Krishnan

**Affiliations:** 1 Department of Mechanical Engineering, Amrita Vishwa Vidyapeetham, Amritapuri, Kollam 690525, India; 2 Department of Foundry Engineering, Silesian University of Technology, Towarowa 7, 44-100 Gliwice, Poland; 3 Department of Mechanical Engineering, Amrita School of Engineering, Amrita Vishwa Vidyapeetham, Coimbatore 641112, India

**Keywords:** A354, fly ash, metal matrix composite, hardness, mechanical properties, automotive application, Al-Si-Mg-Cu-fly ash, A354—fly ash, stir casting/liquid metallurgy route, fractography

## Abstract

The authors researched the physical, metallurgical, and mechanical characteristics of A354 alloy (Al-Si-Mg-Cu) reinforced with 5, 10, and 15 wt% of fly ash metal matrix composites. A baseline alloy and three composites were fabricated by a liquid metallurgy route and poured into a permanent mold to obtain cast rods of dimension Φ32 mm × 156 mm. The metallurgical characterization of the developed alloy and metal matrix composites was conducted using energy-dispersive spectroscopy (EDS), field-emission scanning electron microscopy (FESEM), and X-ray diffraction. All the developed composites showed a pore-free nature, but only A354 alloy reinforced with 5 wt% of fly ash (AF_5_) possessed a homogeneous distribution and perfect bonding of the fly ash with the A354 matrix. Therefore, transmission electron microscopy (TEM) analysis was performed on the sample AF_5_. All developed alloys and metal matrix composites were subjected to hardness and mechanical property tests. It was observed that the AF_5_ sample had 170 ± 5.6 HV and tensile strength of 216 ± 2.3 MPa, 18.8% and 24.8% higher than the A354 matrix, but the ductility (6.5 ± 0.43%) was reduced by 23% from the baseline alloy. Finally, the fractography analysis was conducted on all the samples using FESEM to analyze the fracture mode. The fabricated 5 wt% fly ash-based metal matrix composite showed better mechanical performance than other samples. Hence, sample AF_5_ is suggested for manufacturing components in automotive and structural parts.

## 1. Introduction

Aluminum and its alloys are utilized in numerous applications in the automotive industry. Many manufacturers use these alloys to cast various parts of passenger cars, substituting cast iron in manufacturing cylinder blocks and cylinder heads to reduce weight. Approximately 20% of CO_2_ and other harmful human emissions come from vehicles that affect the world’s ecosystem. The increase in the emission of harmful pollutants, extreme consumption of energy and resources, and ineffective recycling practices significantly affect the environment [1]. In this regard, using Al and its alloys or composites has become essential [2]. Due to financial pressure to reduce harmful CO_2_ emissions and fuel consumption, researchers conduct investigations to minimize the weight of the cars’ structures, and the design has been considerably improved [3,4]. The manufacture of molded automotive parts (particularly cylinder heads) comprises alloys from the 3xx.x series (Al-Si-Mg/A356, Al-Si-Cu, Al-Si-Mg-Cu/A354) [5,6,7,8,9]. These alloys are considered to possess good castability [10,11], relatively low weight, and moderate mechanical properties [5,6,7,8,9]. Approximately 100% of engine pistons, about 80–85% of exhaust manifolds, 70–75% of combustion engine cylinder heads and gearbox housings, and other power transmission components such as drive shafts, rear axles, and differential casings are manufactured in the form of metal casting [2,5,12]. Generally, the cylinder heads are manufactured using permanent mold casting/gravity die casting [6,9,13]. As these Al alloys possess only moderate hardness, strength, and ductility and feeble wear resistance, studies have focused on implementing a single technique or a combination of multiple techniques such as T_6_ heat treatment, surface modification, addition of alloying elements, and reinforcing ceramic particles in the matrix (Metal Matrix Composites) [14]. 

Metal matrix composites (MMCs) acquire considerably better properties, including high mechanical properties, specific modulus, specific strength, damping capacity, and reduced specific wear rate and low density [15,16,17,18]. The reinforcement used in the metal matrix affects the mechanical strength and durability of the developed material compared to the parent material/unreinforced alloy [18]. There has been an emerging trend among the industries in procuring composite materials with low-cost reinforcements and low density [19]. Among several ceramic reinforcements used by different researchers, fly ash is an inexpensive suspended particulate matter obtained as a solid waste by-product during combustion in coal-fired thermal power plants and cement kilns in enormous quantities [20,21,22]. Hence, MMCs with fly ash as reinforcement gain more importance and are expected to overcome the cost obstacle for the extensive application of composite materials in the automotive and aerospace industries. In addition, as part of industrial symbiosis, waste products that cause air pollution can be safely recycled and disposed of. 

MMCs are fabricated by stir casting [18], friction stir processing [23], ball milling [24,25], powder metallurgy, and vacuum hot pressing. Among these techniques, casting is the most popular manufacturing method due to its low cost and high production rate [18]. Research has been conducted on reinforcing various ceramic particles into Al and its alloys to enhance their mechanical and tribological properties. Several authors have investigated the reinforcing of fly ash in the Al-Si-Mg alloy and observed the variation in mechanical and tribological properties [26,27,28]. He et al. [29] developed a composite material to reinforce fly ash nanoparticles to the Al-Si-Mg-Cu alloy by ultrasonic vibration. It was observed that A354/SiC/0.5p-T6 with an additional 15 min of ultrasonic treatment had the highest mechanical properties. Du et al. [30] investigated the effect of SiC on Al-Si-Mg-Cu-Ni through an in situ process. It was observed that the developed composites might be suitable for high-temperature applications. Aybarc et al. [31] presented an overview of reinforcements such as SiC, Al_2_O_3_, and graphene introduced into the aluminum alloy matrix. The research confirmed that the stir casting method is the best way to introduce solid particles into a liquid aluminum alloy matrix. With its outstanding properties, graphene appears to have the potential to become a perfect reinforcement for aluminum alloys. This combination can produce higher toughness with minimal additions compared to ceramic particle additions [31]. Some authors conducted experiments with the carbon nanotube-reinforced aluminum matrix composites, as described in [32]. There has been significant progress in the field of CNT-reinforced aluminum composites. However, due to the problems regarding CNT dispersion and a weak bonding interface, CNT–Al composites are still in the early stage of exploration, and practical applications are still minimal [32]. Another approach was presented in [33], where AMC reinforced with boron nitride nanotubes (BNNT) was described. Again, some problems appear when the particles are to be introduced into the liquid metal matrix. Therefore, different methods were investigated, and it was found that UTS increased by 20% and elongation at fracture was increased by 170% for some combination of treatment parameters [33]. To examine different properties of modern aluminum matrix composites, new methods have been developed, as in [34], where the free vibration analysis was used to investigate the A357 alloy reinforced with dual-particle-size silicon carbide metal matrix composite.

The above discussion shows that most studies are conducted to reinforce fly ash to Al-Si-Mg alloys. No research has been undertaken to strengthen these particles to the A354 alloy. Since Al-Si-Mg-Cu alloy is a promising material for manufacturing cylinder heads in the automobile industry, and fly ash particles in the air harm humanity, recycling these fly ash particles as a reinforcement is worthwhile. Therefore, this current investigation is conducted to enhance the properties of the Al-Si-Mg-Cu alloy by reinforcing varying weight percentages of fly ash and to determine the morphological and mechanical behavior of the developed metal matrix composites.

## 2. Materials and Methods

### 2.1. Fabrication of Metal Matrix Composite

**Figure 1:**
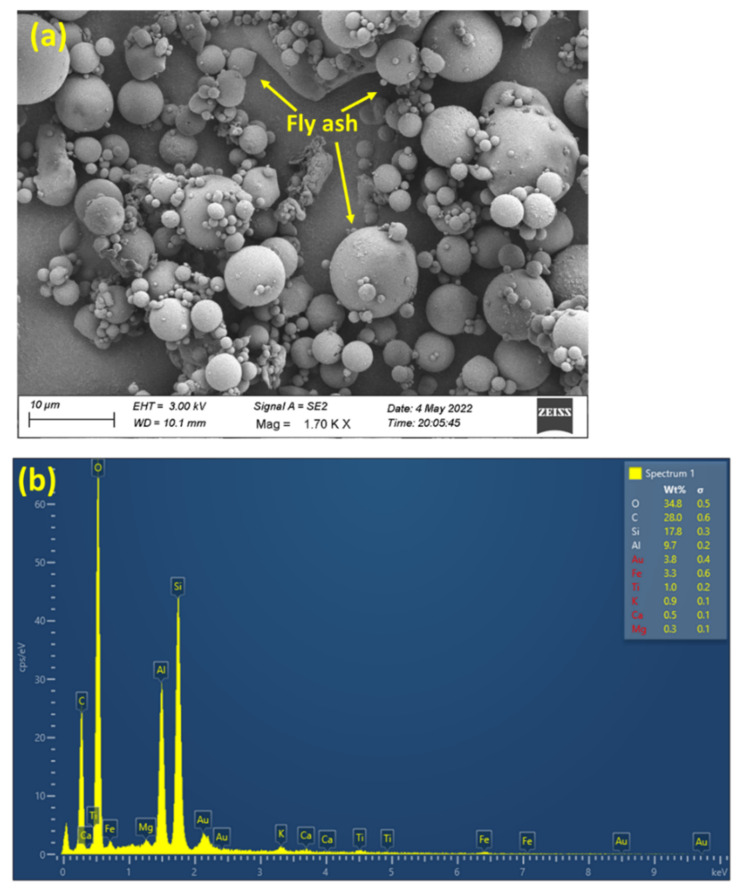
Image showing (**a**) fly ash particles at higher magnification and (**b**) EDS analysis of fly ash.

**Figure 2:**
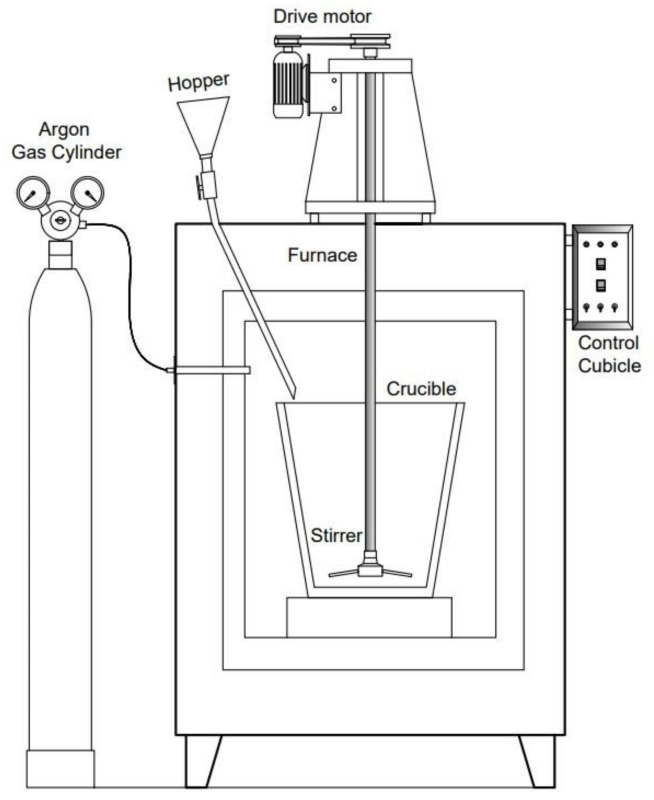
A melting furnace equipped with a mechanical stirrer.

**Figure 3:**
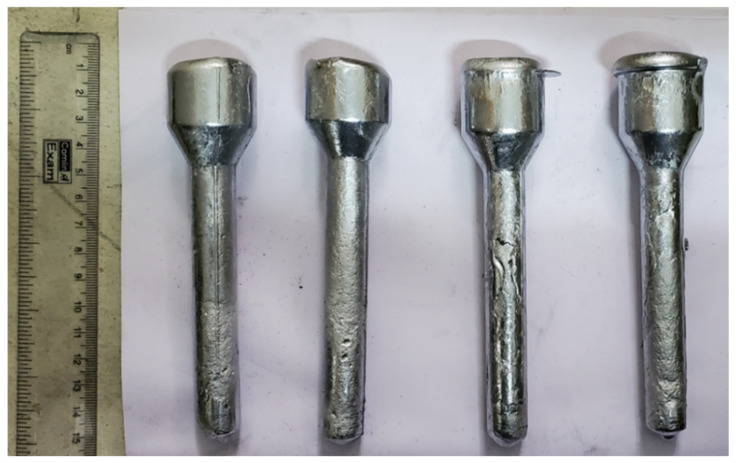
Cast sample rods.

The Al-7Si-3Cu-0.4Mg alloy that belongs to the Al-Si-Mg-Cu group of alloys is used to manufacture cylinder heads and other automotive components [9,30,35,36]. The chemical composition of the alloy investigated in this research is presented in Table 1. The casting manufacturer supplied the alloy melted in an electric furnace with an overheat level of 760–780 °C. The electric furnace was equipped with a mechanical stirrer to obtain a homogeneous distribution of the reinforcement in the matrix. Fly ash with an average particle size of 5 µm (analyzed with Image J software, V 1.8.0. 4.1. (827)), as shown in Figure 1, was preheated in an electric furnace at 300 °C and gradually added to the melt with the support of a hopper. Table 2 depicts the chemical composition of the fly ash particle used in this present investigation. A schematic image of the electric furnace equipped with a mechanical stirrer and hopper is shown in Figure 2. The melting of the composite was conducted in an inert Ar atmosphere to prevent the oxidation of the molten metal. The stirrer was rotated at 200 rpm for 10 min to obtain a uniform distribution of fly ash in the Al matrix. To enhance the wettability of the reinforcement with the matrix, 1 wt% Mg was added to the melt. After proper mixing, the melt was poured into a preheated Lubrikote-coated permanent mold to obtain cast rods of dimensions Φ32 mm × 156 mm, as shown in Figure 3. The procedure mentioned above was followed to obtain MMCs of varying wt% of fly-ash (0, 5, 10, 15). Table 3 shows the details of the materials used in the investigation. As per ASTM standards, the cast specimens were machined to suitable dimensions for morphological and mechanical property testing. Advanced instrumental characterization (FESEM, EDS, TEM, XRD) was used to characterize the specimens.

### 2.2. Density

Using the Archimedes principle, the experimental density values for all samples depicted in Table 4 were calculated. The test was carried out according to the ASTM B962-13 standard. The theoretical density for all samples was calculated using the rule of mixtures. Table 3 shows the values of the experimental and theoretical density. Table 3 shows that the value of the experimental density was found to be lower than the theoretically obtained density. The theoretical density of the fabricated composites (AMMC) was calculated using Equation (1), where ρ _(AMMC)_ is the density of aluminum metal matrix composite, ρ_m_ is the matrix density, ρ_p_ is the particle density, and V_f(m),_ and V_f(p)_ are the volume fraction of matrix and reinforced ceramic particles. Before calculating the theoretical density using the mixtures rule, the alloying elements’ weight percentage was converted into volume fractions.

ρ _(AMMC)_ = ρ_m_ × V_f(m)_ + ρ_p_ × V_f(p)_ (1)

### 2.3. Microstructure and Hardness Test

Scanning electron microscope (SEM) and energy-dispersive spectroscopy (EDS) were used to analyze the metallurgical behavior and composition of the developed alloy and MMCs used in this investigation. Before SEM analysis, the specimens for microstructure and microhardness were prepared as per the standard (American Society for Testing and Materials) ASTM E3-01. The specimens were cut to dimensions of 10 mm × 10 mm. The specimens were mounted using a cold-setting compound. The mounted samples were polished in automatic disc-polishing units with various abrasive sheets (400 grit to 2000 grit). The specimens were then polished in a slurry of diamond particles to achieve a mirror-polished surface. Subsequently, the samples were etched using the standard etchant prescribed in the ASM handbook. Vickers microhardness tester (Mitutoyo, Kawasaki, Japan, Model: HM-210 A) was used to ascertain the microhardness value of the fabricated base alloy (A354) and MMC according to the ASTM E-384 standard. Before testing the hardness value, standard silicon carbide paper grit (emery sheet) was used to polish the surface of the specimens. A load of 1N was applied to the sample’s surface for approximately 30 s. The indentation was applied at five different locations, and an average hardness value for the material was calculated. The above procedure was repeated for all samples shown in Table 2.

### 2.4. Tensile Test

**Figure 4:**
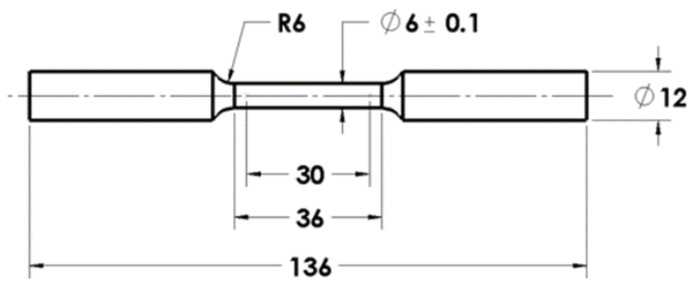
ASTM standard used for machining tensile samples (All dimensions are in mm).

Tensile tests for the base alloy and fabricated MMCs were conducted using a universal testing machine (Tinius Olsen). The samples were machined according to the ASTM E8-04 standard. All samples were machined per the dimensions depicted in Figure 4. Fractography analysis was performed using FESEM analysis to determine the failure mode.

## 3. Results and Discussions

### 3.1. Metallurgical Analysis

**Figure 5:**
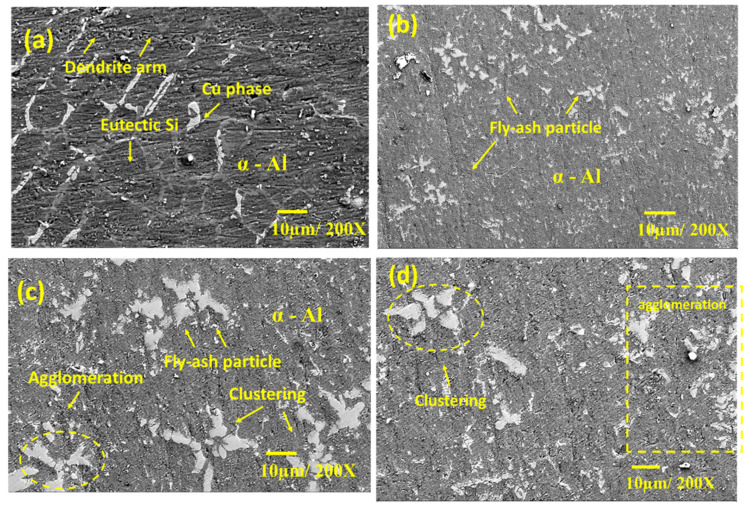
SEM image of the alloy A354 (**a**) as-cast condition (**b**) 5 wt% fly ash (**c**) 10 wt% fly ash (**d**) 15 wt% fly ash.

**Figure 6:**
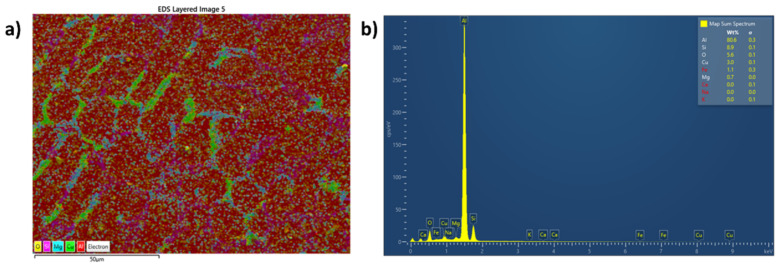
(**a**) Map spectrum (**b**) EDS of A354 alloy in the as-cast condition.

Figure 5a shows the microstructure of A354 alloy in the as-cast condition. The microstructure of A354 in the as-cast state comprises α-Al enriched and eutectic Si phase. In addition, several intermetallic phases were observed in the grain boundaries of the alloy in the as-cast condition. Figure 5b–d show the microstructure of the samples AF_5_, AF_10_, and AF_15,_ respectively. The addition of fly ash to A354 alloy modified the morphology of the eutectic Si phase, as observed in Figure 5b–d. EDS analysis and the elemental mapping of the as-cast A354 alloy are depicted in Figure 6a,b. Figure 5b,c shows the SEM micrograph of the A354 alloy reinforced with 5 wt% and 10 wt% of fly ash reinforcement. It can be seen from the figure that the proper distribution of fly ash is observed in the A354 matrix reinforced with 5 wt% of fly ash. It can also be seen from the figure that the distribution of fly ash particles is, in addition, the same for AF_5_ and AF_10_. These results show the successful fabrication of the metal matrix composite. However, Figure 5c depicts the SEM image of A354 alloy reinforced with 15 wt% fly ash particles (AF_15_). 

**Figure 7:**
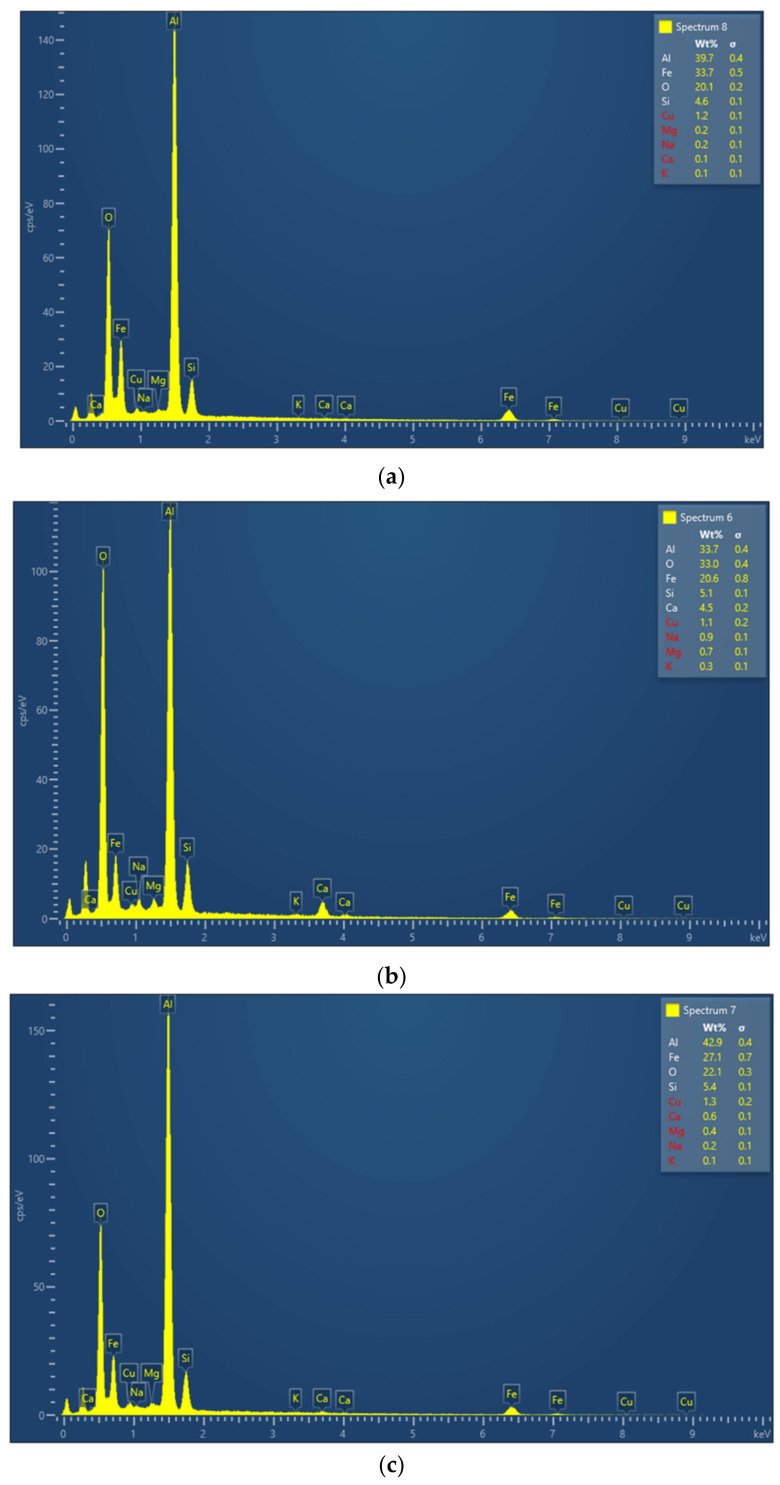
EDS analysis of (**a**) AF_5_; (**b**) AF_10_; (**c**) AF_15_.

**Figure 8:**
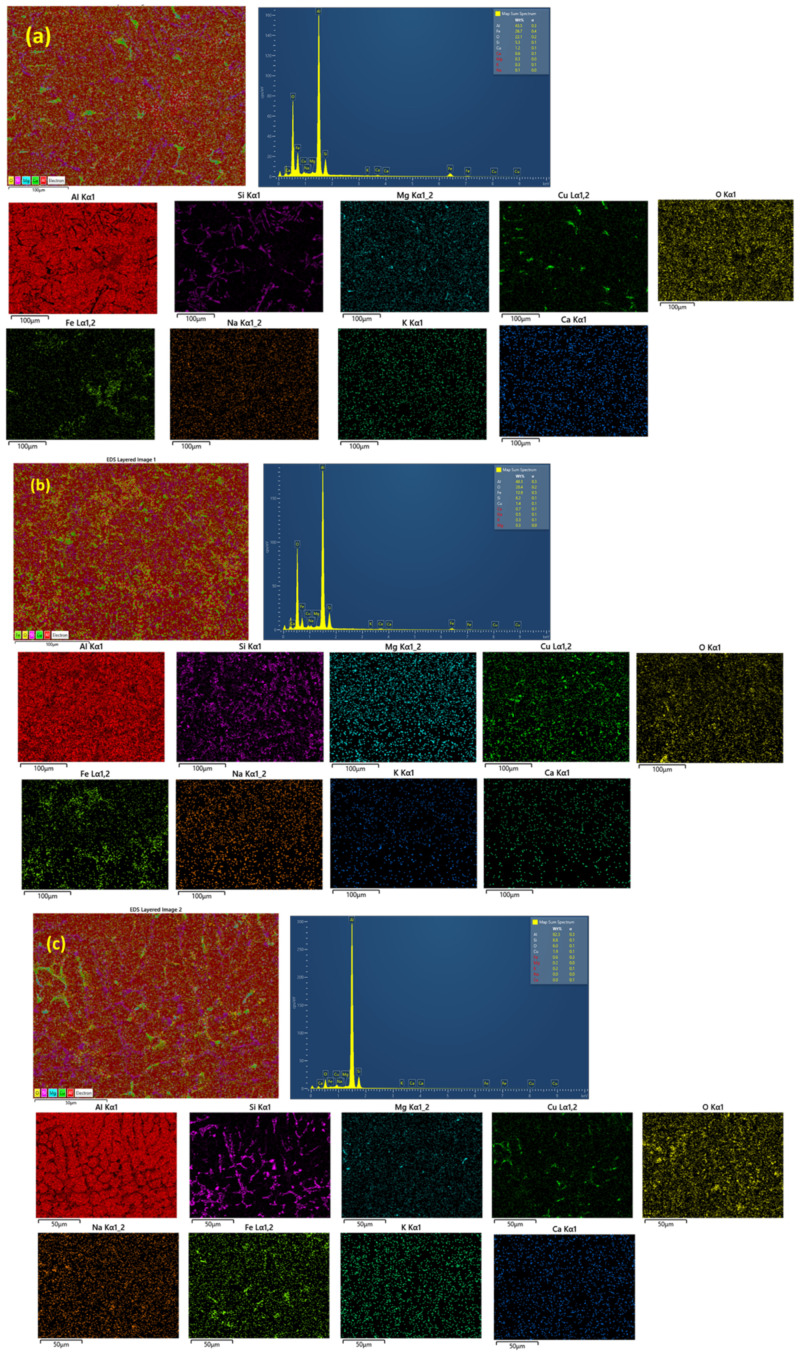
Elemental map spectrum of (**a**) AF_5_; (**b**) AF_10_; (**c**) AF_15_.

It can be observed from Figure 5d that the fly ash particles are in the state of an agglomerated condition within the matrix. Hence, non-homogeneously distributed fly ash particles in this developed MMC would be detrimental to the mechanical behavior [20]. The metal matrix composite can achieve increased mechanical properties when the reinforcement particles are embedded and homogeneously distributed within the matrix. Figure 7 shows the energy-dispersive spectroscopy (EDS) analysis and elemental map spectrum (Figure 8) of all the developed composites to confirm the existence of all elements associated with A354 and its composites. To ensure the presence of alloying elements in A354 alloy, optical arc spectrometry (Foundrymaster, Germany) was used. Five samples were tested, and the average value was calculated. The composition of the A354 alloy is depicted in Table 1. Furthermore, based on the morphological analysis, there was no sign of porosity or defect in the cast samples.

#### 3.1.1. XRD Analysis

**Figure 9:**
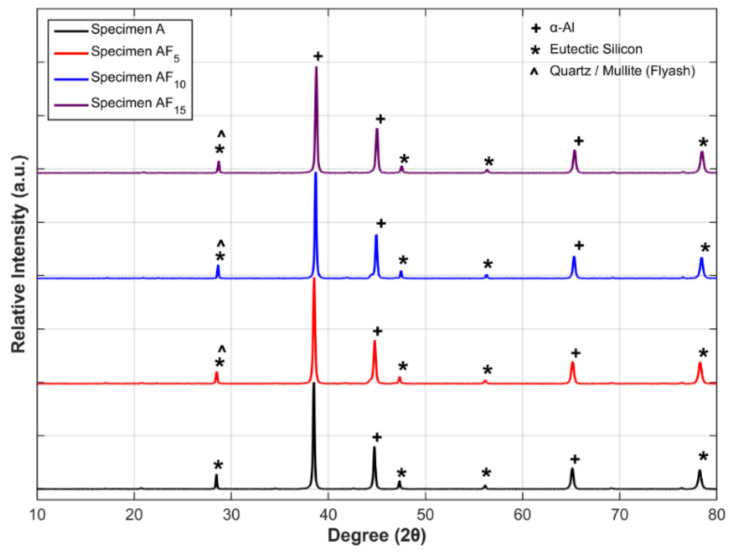
XRD pattern of the base alloy and metal matrix composite material developed.

X-ray diffraction on the A354 and its composite was conducted to determine the presence of fly ash and any intermediate phases. Figure 9 shows the XRD pattern of the fabricated samples in which the A354 alloy specimen showed α-Al and eutectic Si, whereas stir-cast A354/fly ash composite specimens exhibited α-Al, eutectic Si, and Quartz/mullite (fly ash) peaks. The formation of extra peaks is attributed to strengthening the fly ash content in the A354 matrix. The XRD results clearly illustrate the successful fabrication of A354 and its composites.

#### 3.1.2. TEM Analysis for the Sample AF_5_

**Figure 10:**
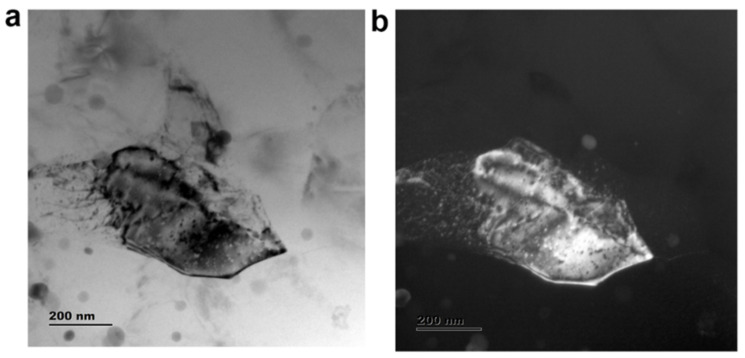
TEM image showing (**a**) bright-field image; (**b**) dark-field image of sample AF_5_.

**Figure 11:**
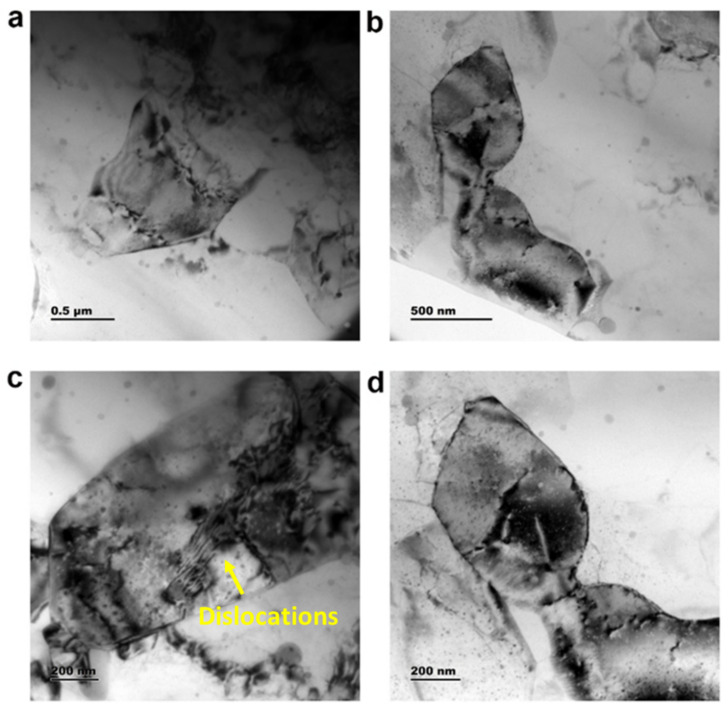
TEM image of the sample AF_5_. (**a**) Refined grains; (**b**) Sub-micron intermetallic phase; (**c**) Dislocation features; (**d**) High magnification image of sub-micron intermetallic phase.

High-resolution transmission electron microscope images of the AF_5_ composite specimen are shown in Figure 10 and Figure 11. The bright-field image depicted the α-Al phase (bright region), eutectic Si (grey region), and submicron fly ash particle (dark region), as shown in Figure 10a. The dark-field image is shown in Figure 10b, contrasted with fly ash (bright region) and eutectic Si (grey region). TEM analysis confirmed the presence of submicron and nano-level eutectic Si in the fabricated composite. Figure 11a–d shows a few dislocations features adjacent to fly ash particles, apart from the fly ash particles and the eutectic Si phase. Furthermore, the authors observed characteristic partial dislocations in Figure 11c. The TEM observation corroborates ceramic particle clustering for the sample AF_5_.

### 3.2. Mechanical Behavior of A354 Alloy and Stir Cast MMC

**Figure 12:**
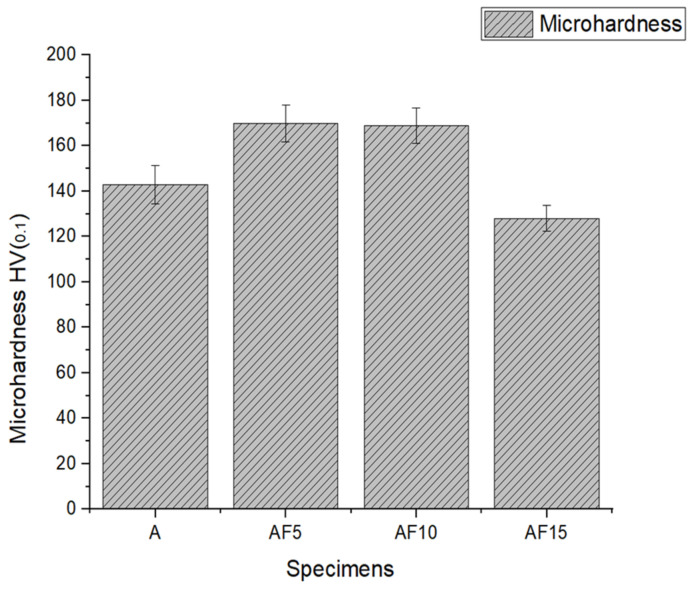
Microhardness of A354 and its composites.

The mechanical characteristics were conducted using Vickers hardness, ultimate tensile strength, yield strength, and ductility (Table 5). With reinforcing varying wt% of fly ash particles, the resulting value of tensile strength, hardness, and yield strength increases from the baseline alloy to MMC reinforced with 5 wt% of fly ash and further decreases to 10, 15 wt% fly ash-reinforced MMC. Figure 11 shows the average Vickers hardness values for all samples with error bars. The resulting measured average hardness was found to be: sample A = 143 ± 5.1 HV, AF_5_ = 170 ± 5.6 HV, AF_10_ = 164 ± 5.5 HV, AF_15_ = 128 ± 5.6 HV, respectively. Sample AF_5_ was shown to be around 18.8%, 3.6%, and 32.8% higher than samples A, AF_10_, and AF_15_. Vickers hardness improved due to the proper mixing and embedding of fly ash particles with the A354 matrix (Figure 12) [1].

Furthermore, a lower fly ash content could have led to more grain refinement with dislocations and efficient bonding of fly ash with the A354 matrix, which had resulted in an increase in the hardness value for sample AF_5_ (Table 4). In addition, the applied load is transformed into the reinforcing particle [2,37,38,39,40,41]. Reinforcing particles withstand the load and geometrically constrain the plastic deformation, which generates the dislocation required to support the load. These dislocations promote the dislocation density of the MMC, causing dislocation hindering. Therefore, the hardness of the MMC increased from the baseline alloy (sample A) to sample AF_5_. The hardness value improved due to the dispersion hardening and Orowan strengthening. In addition, more resistance against plastic deformation was anticipated from the fly ash particle because of the hard surface area, resulting in the fabrication of pore-free surfaces. The presence of agglomeration caused by a nonuniform mix of the reinforcement particles on the A354 matrix led to a lower hardness value in 10 wt%, and 15 wt% fly ash reinforced MMC (Figure 5c,d).

**Figure 13:**
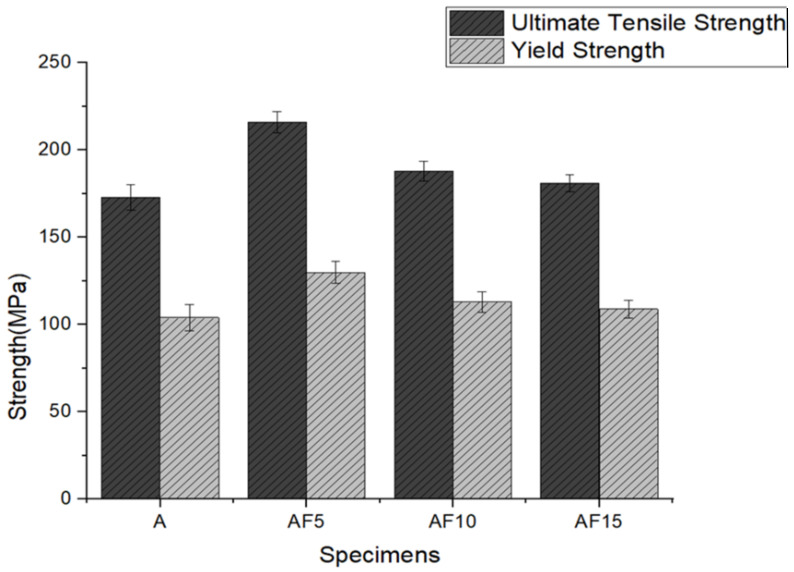
Tensile and yield strength of A354 and its composites.

Figure 13 shows the average tensile and yield strength for fabricated alloy and MMCs. The average UTS values, yield strength, and standard deviation for the baseline alloy and MMCs are depicted in Table 5. The A354 alloy showed an ultimate tensile strength of 173 MPa and yield strength of 104 MPa, and the alloy reinforced with varying wt% of fly ash possesses higher UTS and yield strength values than the base alloy. The highest UTS was shown by sample AF_5_ (216 MPa), which is 24.8% higher than the value indicated by sample A (173 MPa), 14.9% higher than sample AF_10_ (188 MPa), and 19.3% higher than sample AF_15_ (181 MPa). Likewise, the higher yield strength was also shown by sample AF_5_ (130 MPa), which is 25% more than the value indicated by sample A, 15% more than sample AF_10_, and 19.26% more than the alloy reinforced with sample AF_15_. Compared with sample A, the improved bonding strength of the reinforcement particle with the alloy improves both the composites’ UTS and yield strength. Moreover, the increase in value of UTS and YS from sample A to sample AF_5_, AF_10_, and AF_15_ is attributed to the dispersion-hardening mechanism. The transfer of tensile load from the alloy to reinforcement particles with higher load capacity delays fracture occurrence in the specimens. The high wettability of fly ash particles in sample AF_5_ enables load transmission and distribution from matrix to reinforcement. Particulate reinforcements have a high elastic modulus, which decreases strain to failure. The increase in tensile strength is attributed to the rise in the volume fraction of reinforcements in the sample AF_5_ compared to the unreinforced alloy.

Consequently, crack formation and propagation can be minimized. The grain size and particle-dispersion capacity determine the yield stress. The low values for the AF_10_ and AF_15_ are due to the agglomeration and clustering caused by the nonuniform mix of the reinforcement particles with the A354 matrix. 

### 3.3. Ductility (Percentage Elongation)

**Figure 14:**
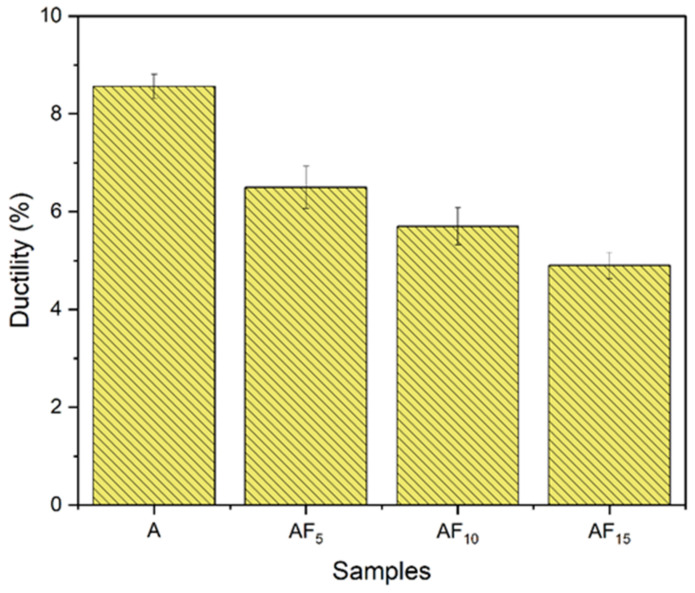
Percentage elongation value of A354 and its composites.

Figure 14 shows the average ductility value of Al-Si-Mg-Cu and its composites. It can be noted from the figure that the average ductility of the base material was 8.5%. It was observed that an increase in the concentration of fly ash decreased the ductility of the specimens. The lowest ductility of 4.9% was observed in specimens with 15 wt% fly ash. The decrease in ductility could be correlated to the concentration of reinforcement particles. Though reinforcement particles augmented the hardness and strength of the material, their inability to yield induced brittle tendency in the specimens. The clustered particles acted as crack initiation sites at a high concentration of fly ash particles and promoted crack growth. Hence, the samples with a high concentration of fly ash had characteristic ductile and brittle features (primary). The results align with the earlier research work [42,43,44].

### 3.4. Fractography

**Figure 15:**
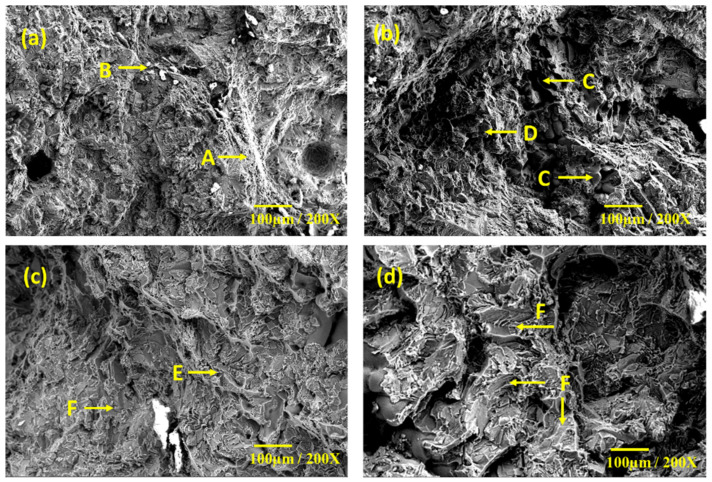
Fractography of A354 alloy. (**a**) As-cast condition; (**b**) 5 wt% fly ash; (**c**) 10 wt% fly ash; (**d**) 15 wt% fly ash.

The fractography of the A354 in the as-cast condition (Figure 15a) exhibited plastic deformation characteristics (marked as A) and cracks (marked as B). However, characteristic equiaxed dimples indicating the ductile nature of the specimen were not observed. The specimens exhibited more brittle fracture features with an increase in fly ash concentration. Typical fractography of the samples AF_5_, AF_10_, and AF_15_ are shown in Figure 15b–d, respectively. The AF_5_ sample exhibited cambers (marked C) and dimples (marked D), indicative of ductility. Moreover, a larger plastic deformation zone was observed in the specimen AF_5_ than in the A354 alloy. In addition, shear dimples and a particle pullout region (large crater) were observed. The AF_10_ specimen exhibited a higher fraction of facets (marked F) and cracks (marked as E) on the fractured surface. The grain facets appear dark, clean, and without any striation marks. The features confirm the intergranular rupture of the specimen during the tensile test. The fractograph of the specimen AF_15_ exhibited typical transcrystalline cleavage, with an array of river patterns decorating the large facets. Furthermore, shear steps and cleavage steps were more prominent in the specimen. Therefore, the AF_15_ specimen had fractured predominantly by a brittle fracture mechanism. The presence of a more significant fraction of hard-phase fly ash (FA) particles increased hardness (or decreased hardness attributed to FA agglomeration), decreased tensile strength, and promoted brittle fracture. The fractograph substantiates the fracture transition from a combined ductile-brittle mechanism towards a predominantly brittle mechanism, with an increase in FA composition.

## 4. Conclusions

A354 alloy-based composite was successfully fabricated via stir-casting technique with varied weight compositions of fly ash (5%, 10%, and 15%). The microstructural evolution, microhardness, tensile properties, and fracture mechanism of the as-cast and fabricated composite samples were tested. The results demonstrated the following:

1. The stir-casting technique fabricated sound castings of A354 alloy and composites.
2. The microstructure of the composite specimen AF_5_ exhibited a homogeneous dispersion of FA particles and a fragmented eutectic Si phase. However, AF_10_ and AF_15_ specimens pronounced a greater agglomeration of FA particles. Furthermore, the AF_15_ sample had preferential segregation of eutectic Si and FA particles along the grain boundaries.
3. The refined microstructure increased the microhardness (~20%), tensile strength (~25%), and yield strength (~25%) of the composite specimen AF_5_ than the A354 alloy. The specimens AF_10_ and AF_15_ had diminished mechanical properties because of heterogeneous microstructure.
4. With an increase in the concentration of FA particles, the fracture mode transformed from a combined ductile-brittle mechanism to a predominantly brittle mechanism in the specimens.

## Figures and Tables

**Table 1:** Chemical composition of the base alloy used in the study.

Elements	Al	Si	Cu	Mg
wt%	Rem.	6.9	2.8	0.39

**Table 2:** Chemical composition of the fly-ash particle.

Elements	Al	Si	O	C	Fe	Others (Na, Mg, K, P, Ca)
wt%	9.7	17.8	34.8	2.8	3.3	Rem.

**Table 3:** Details of the samples used in the investigation.

Sample Code	Matrix	Reinforcement	Wt% Reinforcement
A	A354	-	-
AF_5_	A354	Fly ash	5
AF_10_	A354	Fly ash	10
AF_15_	A354	Fly ash	15

**Table 4:** Density value of A354 and its composites.

Sl No	Samples	Density Values in g/cm^3^	
		Experimental	Theoretical
1	A	2.75 ± 0.014	2.79
2	AF_5_	2.45 ± 0.013	2.53
3	AF_10_	2.42 ± 0.012	2.48
4	AF_15_	2.39 ± 0.011	2.41

**Table 5:** Mechanical properties of baseline alloy and stir-cast A354 reinforced with fly ash particles.

Samples	Hardness (HV_0.1_)	Ultimate Tensile Strength (MPa)	Yield Strength (MPa)	Ductility (%)
A	143 ± 5.1	173± 2.1	104 ± 3.2	8.5 ± 0.25
AF_5_	170 ± 5.6	216 ± 2.3	130 ± 2.2	6.5 ± 0.43
AF_10_	164 ± 5.5	188 ± 2.2	113 ± 3.2	5.7 ± 0.38
AF_15_	128 ± 5.6	181 ± 2.1	109 ± 2.1	4.9 ± 0.27

## Data Availability

The data presented in this study are available on request from the corresponding author.
